# Importance of formalin fixing conditions for HER2 testing in gastric cancer: immunohistochemical staining and fluorescence in situ hybridization

**DOI:** 10.1007/s10120-013-0329-8

**Published:** 2014-01-12

**Authors:** Yoriko Yamashita-Kashima, Sei Shu, Keigo Yorozu, Kaoru Hashizume, Yoichiro Moriya, Kaori Fujimoto-Ouchi, Naoki Harada

**Affiliations:** 1 Product Research Department, Chugai Pharmaceutical Co., Ltd, 200, Kajiwara, Kamakura, Kanagawa 247-8530 Japan; 2 Medical Plan Management Department, Chugai Pharmaceutical Co., Ltd., 2-1-1, Nihonbashi-Muromachi, Chuo-ku, Tokyo, 103-8324 Japan

**Keywords:** Gastric cancer, HER2 test, IHC, FISH, Formalin fixation

## Abstract

**Background:**

Accurate and reliable assessment of human epidermal growth factor receptor type 2 (HER2) status is important for selecting patients with gastric cancer who may benefit from trastuzumab treatment. Here we examined the impact of formalin fixing conditions on HER2 immunohistochemistry (IHC) and fluorescence in situ hybridization (FISH) in xenografted tumor tissues.

**Methods:**

Xenografted tumor tissues of the human gastric cancer cell lines NCI-N87, SCH, and SNU-16 were collected and kept at room temperature for 0, 6, or 24 h before being fixed with 10 % neutral buffered formalin (NBF) for 24 h or 5, 7, or 10 days and embedded in paraffin. Use of 10 % NBF, 20 % NBF, or nonbuffered formalin as fixative was investigated.

**Results:**

The HER2 IHC scores for NCI-N87, SCH, and SNU-16 tumors were 3+, 2+, and 1+, respectively, when specimens were fixed with 10 % NBF for 24 h immediately after resection of the tumors. Specimens left for longer than 6 h before fixation had shrinkage of the tumor periphery and decreased immunostaining intensity in this region in all specimens. In SCH and SNU-16 specimens, starting fixation 24 h after tumor tissue collection induced autolysis and reduction of the number of stained cells, and 10-day-fixation lowered the HER2 score. Prolongation of fixation time did not affect FISH results, but if samples were left for more than 6 h before fixation, the FISH score was strongly reduced in SCH specimens (2.3 to 1.3). Reduced IHC staining intensity was observed with 20 % NBF and nonbuffered formalin compared to 10 % NBF.

**Conclusions:**

The time to and length of fixation of tumor specimens can affect HER2 IHC and FISH scores. The fixative used can affect IHC results.

## Introduction

Gastric cancer is the forth frequently diagnosed tumors in the world with 989,000 cases estimated to have occurred [1] and is the second leading cause of cancer death worldwide (738,000 deaths; 9.7 % of all cancers) as of 2008 [1]. Although fluoropyrimidine- or platinum-based combination chemotherapy are the most widely accepted regimens for advanced gastric cancer in the world at present, their benefit has not necessarily been translated into higher overall survival rates. The addition of the HER2-targeted agent trastuzumab to these standard chemotherapy regimens has been investigated for patient with HER2-positive disease. HER2, a member of the HER family proteins, regulates cell proliferation, differentiation, and apoptosis. These functions are triggered by the formation of HER2 homodimers or heterodimers with other HER family proteins [2] and subsequent activation of downstream signaling. In breast cancer, HER2 overexpression is a prognostic factor [3–5] and an important predictive marker for determining which patients are likely to benefit from treatment with trastuzumab. In gastric cancer, there is controversy concerning whether HER2 status provides prognostic information [6]. However, it is necessary to determine HER2 status to determine whether trastuzumab will be a beneficial option for patients.

The ToGA trial, an open-label, international, phase III, randomized controlled trial, demonstrated that patients with HER2-positive gastric cancer assigned to receive trastuzumab plus standard chemotherapy had significantly longer overall survival (OS) (hazard ratio: HR 0.74, 95 % CI, 0.60–0.91) compared with patients with HER2-positive gastric cancer assigned to receive chemotherapy alone [7]. The improvement in OS in the trastuzumab arm was more apparent in patients whose tumors had high HER2 expression [immunochemistry (IHC) 3+, or IHC 2+ and fluorescence in situ hybridization (FISH) positive], with a HR of 0.65 (95 % CI, 0.51–0.83). Based on the results of the ToGA study, regimens that include trastuzumab plus chemotherapy became a new standard treatment modality for HER2-positive gastric cancer.

Immunohistochemistry analysis, which evaluates protein expression, and FISH analysis, which evaluates gene amplification, are major methods for HER2 testing. Although assessing HER2 status accurately and reliably is of great importance to determine whether patients have HER2-positive breast or gastric cancer, there is variability of sample preparation for HER2 testing [8]. A number of reports show discordance in HER2 testing results between laboratories [9, 10]. Perez et al. [11] have shown the importance of using high-volume, experienced laboratories for HER2 testing to improve the process of selecting patients likely to benefit from trastuzumab therapy. To ensure accurate evaluation, the American Society of Clinical Oncology (ASCO) and College of American Pathologists (CAP) implemented guidelines for HER2 evaluation in breast cancer in 2007 [12].

By virtue of the ASCO/CAP guidelines, the method of preparing specimens has been standardized in breast cancer. In Japan, HER2 testing guidelines for gastric cancer were defined by the trastuzumab pathological advisory board for gastric cancer in line with ASCO/CAP guidelines. Paraffin sections are prepared, and the IHC scoring system is used according to these criteria that aimed to develop a validated HER2 scoring system for gastric cancer [13]. However, interlaboratory differences in the results of HER2 testing are still a considerable problem in gastric cancer, and one cause may be inappropriate preparation of formalin-fixed paraffin-embedded tissues. In the present study, we investigated the effect of formalin fixing conditions, including time to fixation, fixation time, and composition of fixatives, on IHC and FISH for HER2 using human gastric cancer cell line xenografted tumor tissues with different HER2 status.

## Materials and methods

### Animals

Five-week-old male BALB-nu/nu mice (CAnN.Cg-Foxn1<nu>/CrlCrlj nu/nu) were obtained from Charles River Laboratories Japan (Yokohama, Japan). All animals were housed in a pathogen-free environment under controlled conditions (temperature 20–26 °C, humidity 40–70 %, light:dark cycle 12/12 h). Chlorinated water and irradiated food were provided ad libitum. All animals were allowed to acclimatize and recover from shipping-related stress for 1 week before the study. The health of the mice was monitored by daily observation. All animal experiments were conducted in accordance with the Guidelines for the Accommodation and Care of Laboratory Animals promulgated in Chugai Pharmaceutical Co., Ltd.

### Cell lines and culture conditions

Three human gastric cancer cell lines were used in the present study. NCI-N87 and SNU-16 cells were purchased from American Type Culture Collection (ATCC) (Rockville, MD, USA). SCH cells were purchased from Japan Health Sciences Foundation (Osaka, Japan). NCI-N87, SNU-16, and SCH were maintained in RPMI-1640 (Sigma-Aldrich, St. Louis, MO, USA) supplemented with 10 % (v/v) fetal bovine serum at 37 °C under 5 % CO_2_. The histological type of each cell line is shown in Table 1.

**Table 1:** Histological type and HER2 status of three gastric tumor tissues

Tumor name	Histological type^a^	HER2 IHC score	HER2/CEP17 ratio (FISH)
NCI-N87	Differentiated/intestinal-type epithelial carcinoma	3+	8.4 [11]
SCH	Undifferentiated-/diffuse-type choriocarcinoma	2+	2.3
SNU-16	Undifferentiated-/diffuse-type epithelial carcinoma	1+	1.4 [11]

^a^Histological type was described according to the information from each cell line distributor

### Preparation of specimens

Each mouse was inoculated subcutaneously into the right flank with 5 × 10^6^ cells/mouse of a human gastric cancer cell line, either NCI-N87, SCH, or SNU-16. Tumor xenograft tissues were collected and allowed to stand at room temperature for 0, 6, or 24 h before being fixed with 10 % neutral buffered formalin (NBF) for 24 h, or 5, 7, or 10 days, and then embedded in paraffin. To compare the effect of fixing solution on HER2 IHC, 10 % NBF, 20 % NBF, 10 % nonbuffered formalin, or 20 % nonbuffered formalin was used. Specimens were prepared from two separate tumors from mice inoculated with NCI-N87 and SNU-16 and three tumors from SCH-inoculated mice.

### IHC, gene amplification of HER2, and histological assessment

HER2 protein expression and HER2 gene amplification in tumors were examined by IHC using HercepTest (Dako, Glostrup, Denmark) and by FISH using PathVysion HER2 DNA Probe (Abbott Molecular, Abott Park, IL, USA), respectively, at SRL (Tokyo, Japan) as described previously [14]. Histological assessment was performed under a light microscope.

## Results

### IHC and FISH status of tumor tissues used

Three tumor tissues with different HER2 status were used in this study. The HER2 status of these tumor tissues were assessed by IHC and FISH, using specimens fixed with 10 % NBF for 24 h immediately after resection of the tumors. The IHC scores for NCI-N87, SCH, and SNU-16 were 3+, 2+, and 1+, respectively, and HER2/CEP17 ratios by FISH were 8.4, 2.3, and 1.4, respectively (Table 1).

### Effect of time to fixation and fixation time on IHC staining for HER2

**Fig. 1:**
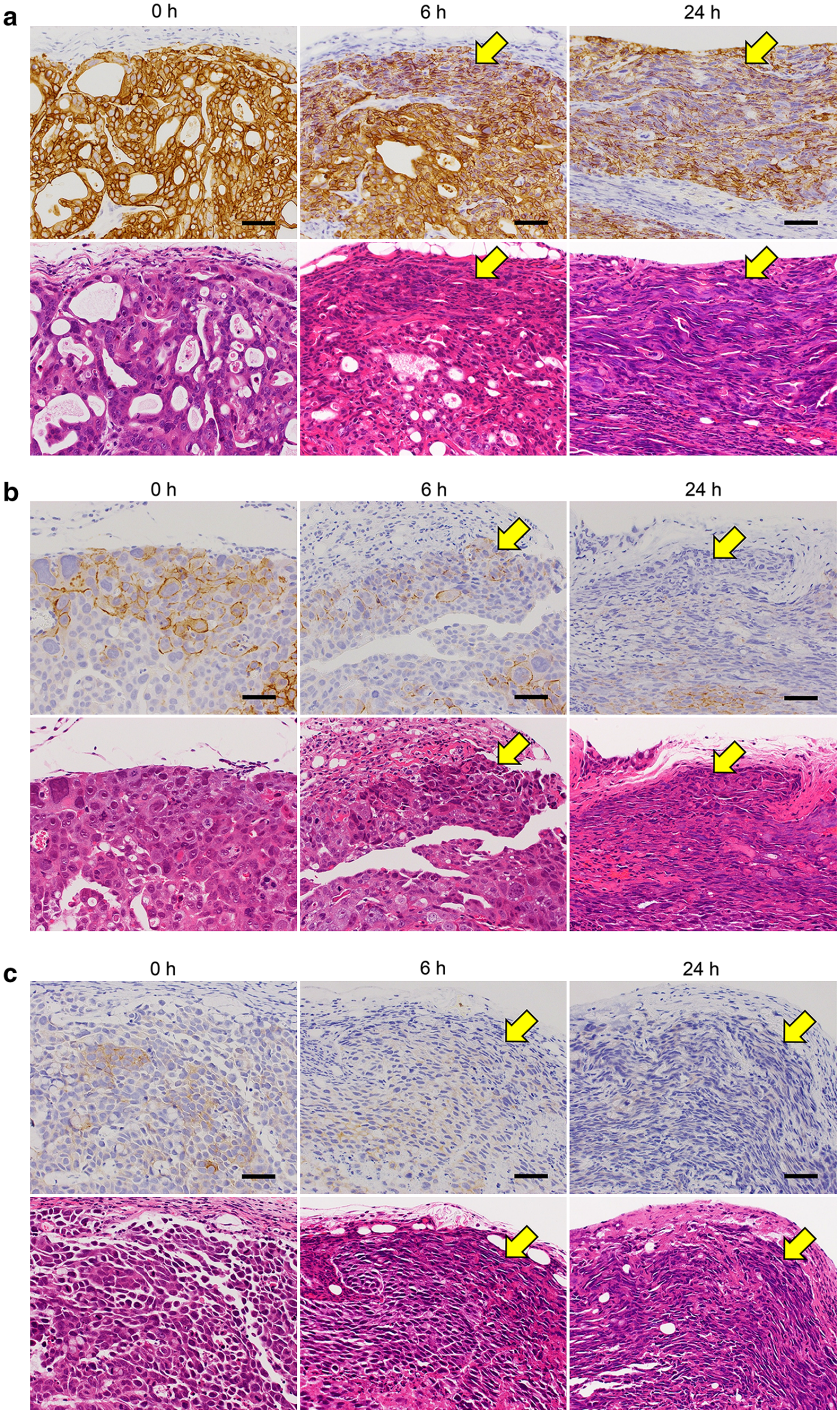
Effect of time to fixation on immunohistochemistry (IHC) staining for HER2 in tumor tissue periphery. Tumor tissue specimens of NCI-N87 (**a**), SCH (**b**), or SNU-16 (**c**) were collected and allowed to stand for 0, 6, or 24 h before fixing with 10 % neutral buffered formalin (NBF) for 24 h. *Upper panels* HER2 IHC staining; *lower panels* hematoxylin and eosin staining. *Arrows* indicate shrunken areas and areas of decreased staining intensity. *Bars* 50 μm

**Fig. 2:**
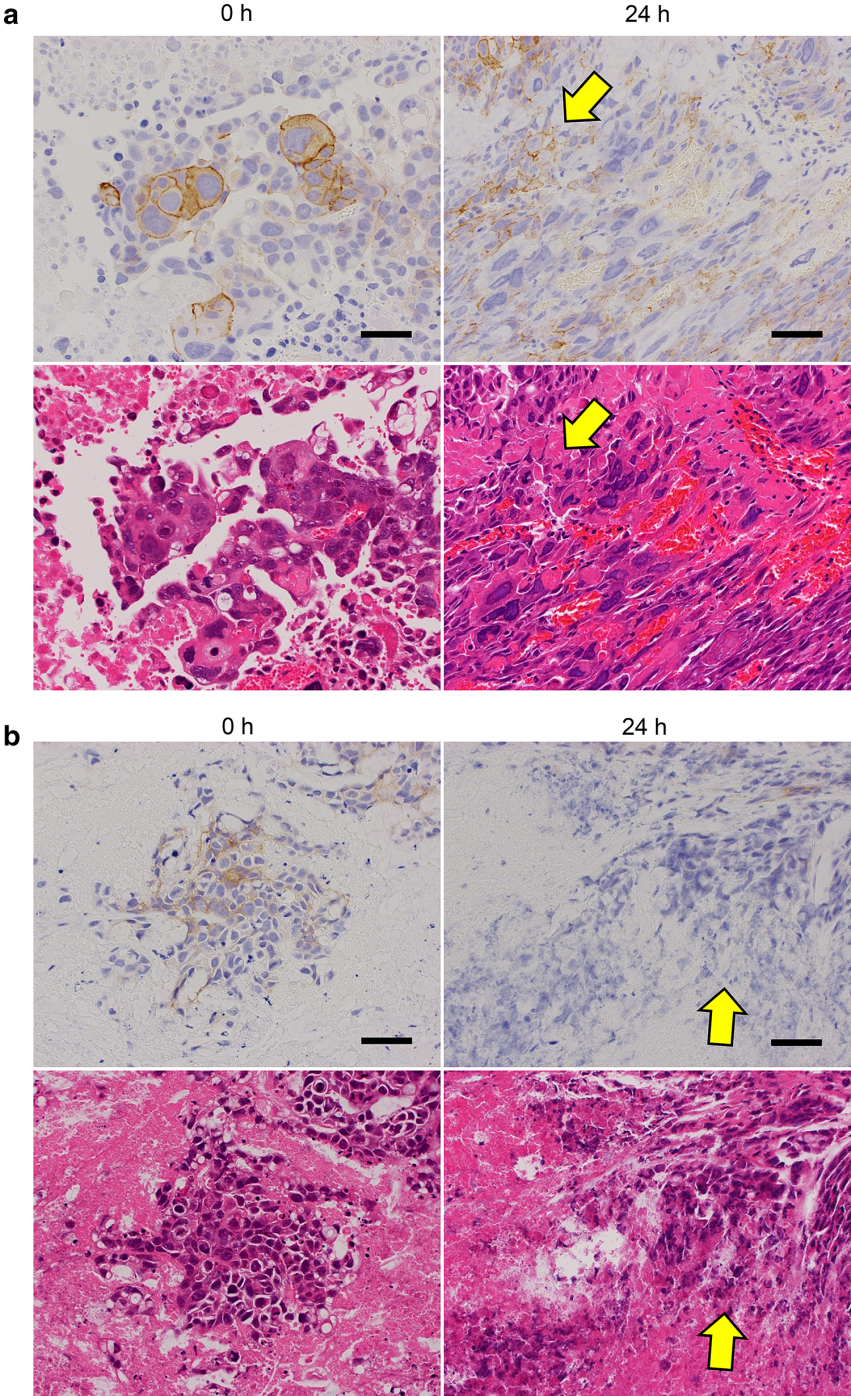
Effect of time to fixation on IHC staining for HER2 in tumor tissue center. Tumor tissue specimens of SCH (**a**) and (**b**) SNU-16 were collected and allowed to stand for 0 or 24 h before fixing with 10 % NBF for 24 h. *Upper panels* HER2 IHC staining; *lower panels* hematoxylin and eosin staining. *Arrows* indicate areas of advanced autolysis. *Bars* 50 μm

First, we examined the effect of time to fixation and length of fixation on IHC staining for HER2 in NCI-N87, SCH, and SNU-16 tumor tissues. Leaving specimens for 6 or 24 h before fixation at room temperature promoted shrinkage in the tumor tissue periphery and decreased immunostaining intensity at the shrinkage area in tumors from all the cell lines, irrespective of HER2 status and fixation time (Fig. 1a–c; Tables 2, 3). In SCH or SNU-16 tumors, leaving the specimens for 24 h before fixation also induced autolysis and decreased the number of stained cells in the center of tumor tissues (Fig. 2a, b; Table 3). As a result, the total staining intensity and total staining area were decreased in these two tumors. In SCH tumors, a decrease of the HER2 IHC score to 1+ was observed in two of three specimens left for 24 h and then fixed for 24 h (Table 2).

**Table 2:** Effect of fixation conditions on HER2 immunochemistry (IHC) score in human breast cancer xenografted tumors

Time to fixation	NCI-N87				SCH				SNU-16			
	Fixation time				Fixation time				Fixation time			
	24 h	5 days	7 days	10 days	24 h	5 days	7 days	10 days	24 h	5 days	7 days	10 days
0 h	3+	3+	3+	3+	2+^a^	2+^a^	2+^a^	1+/2+	1+	1+	1+	0/1+
6 h	3+	3+	3+	3+	2+	2+/3+	2+	2+	1+	1+	1+	0/1+
24 h	3+	3+	3+	3+	1+/2+	1+^a^	1+/2+	1+/2+	1+	0/1+	1+	0

HER2 IHC was performed by HercepTest

Tests were performed in duplicate for NCI-N87 and SNU-16, in triplicate or duplicate (a) for SCH. If the test results of duplicate samples were different, both values were indicated

**Table 3:** Effect of fixation conditions on the detailed histological assessment of HER2 IHC staining in human breast cancer xenografted tumors

Time to fixation	NCI-N87				SCH				SNU-16			
	Fixation time				Fixation time				Fixation time			
	24 h	5 days	7 days	10 days	24 h	5 days	7 days	10 days	24 h	5 days	7 days	10 days
0 h	–	–	–	–	–^g^	–^g^	–^g^	–/+	–	+	+	+/++
6 h	±	±	±	±	–/±	±	–/±	–/±	±	±/+	+	+/++
24 h	±	±/+	±/+	±	+	+^g^	±/+	±/+	+	+/++	+	++/+++

Histological assessment of decrease in staining was performed in terms of the following criteria and judged in a comprehensive manner: (a) tumor periphery shrinkage (dry), (b) decrease of staining intensity at shrinkage area, (c) autolysis, (d) decrease in positive cells, (e) decrease of total staining intensity, (f) decrease of total staining area

Assessments were performed in duplicate for NCI-N87 and SNU-16, in triplicate or duplicate (g) for SCH. If the results of duplicate or triplicate samples were different, all results were indicated

–, no change; ±, very slightly decreased; +, slightly decreased; ++, moderately decreased; +++, markedly decreased

**Fig. 3:**
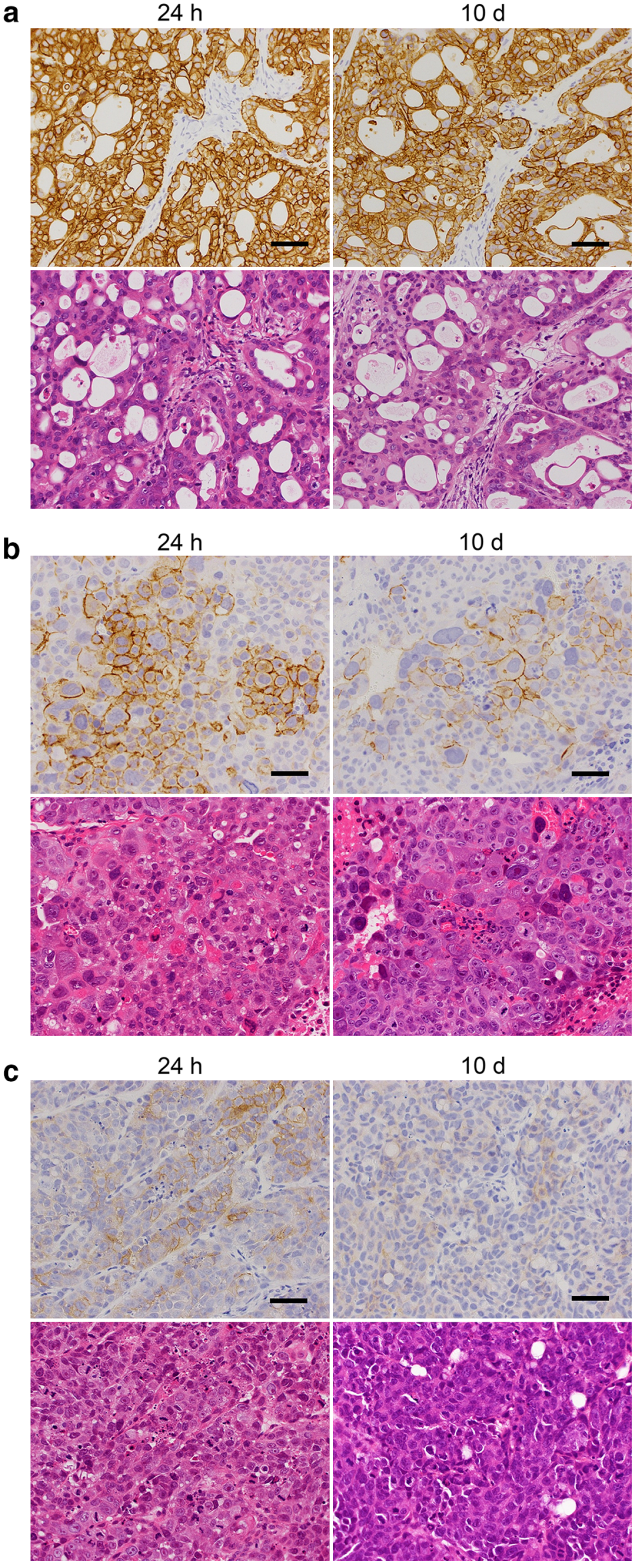
Effect of fixation time on IHC staining for HER2. Tumor tissue specimens of NCI-N87 (**a**), SCH (**b**), and SNU-16 (**c**) were collected and immediately fixed with 10 % NBF for 24 h or 10 days. *Upper panels* HER2 IHC staining; *lower panels* hematoxylin and eosin staining. *Bars* 50 μm

Second, we examined the effect of fixation time on IHC staining for HER2. In NCI-N87 specimens from tumors with a HER2 IHC 3+ score, there was no change in HER2 IHC score (Fig. 3a; Table 2). However, in SCH or SNU-16 specimens from tumors with HER2 IHC 2+ or 1+ scores, the scores were decreased to 1+ or 0, respectively, in one of three or one of two specimens by fixing for 10 days, even when fixation was started immediately after the tumor collection (Fig. 3b, c; Table 2). Moreover, in SNU-16 tumor specimens that were left for 24 h before fixation, prolonged fixation time (10-day fixation) promoted the decrease of HER2 staining intensity at the shrunken area and a decrease in HER2-positive cells (Table 3). HER2 IHC scores for these specimens were 0 in all specimens (two of two) (Table 2).

### Effect of time to fixation and fixation time on FISH results

We also investigated whether delay to formalin fixation or prolonged fixation time affects FISH results. SCH specimens in which time to fixation and fixation time were (1) 0 and 24 h, (2) 0 h and 10 days, (3) 6 and 24 h, (4) 24 and 24 h, or (5) 24 h and 10 days were assessed by FISH (HER2/CEP17 ratio). The mean of the HER2/CEP17 ratios of specimens of times (1), (2), (3), (4), and (5) were 2.3, 2.6, 1.3, 1.2, and 1.1, respectively, suggesting that prolonged fixation time did not affect the FISH results but that delay to fixation strongly reduced the FISH score (Table 4).

**Table 4:** Effect of fixation conditions on HER2 FISH in SCH xenografted tumors

Time to fixation	Fixation time	HER2/CEP17 ratio (FISH)
0 h	24 h	2.3
0 h	10 days	2.6
6 h	24 h	1.3
24 h	24 h	1.2
24 h	10 days	1.1

### Effect of fixative type on IHC staining for HER2

**Fig. 4:**
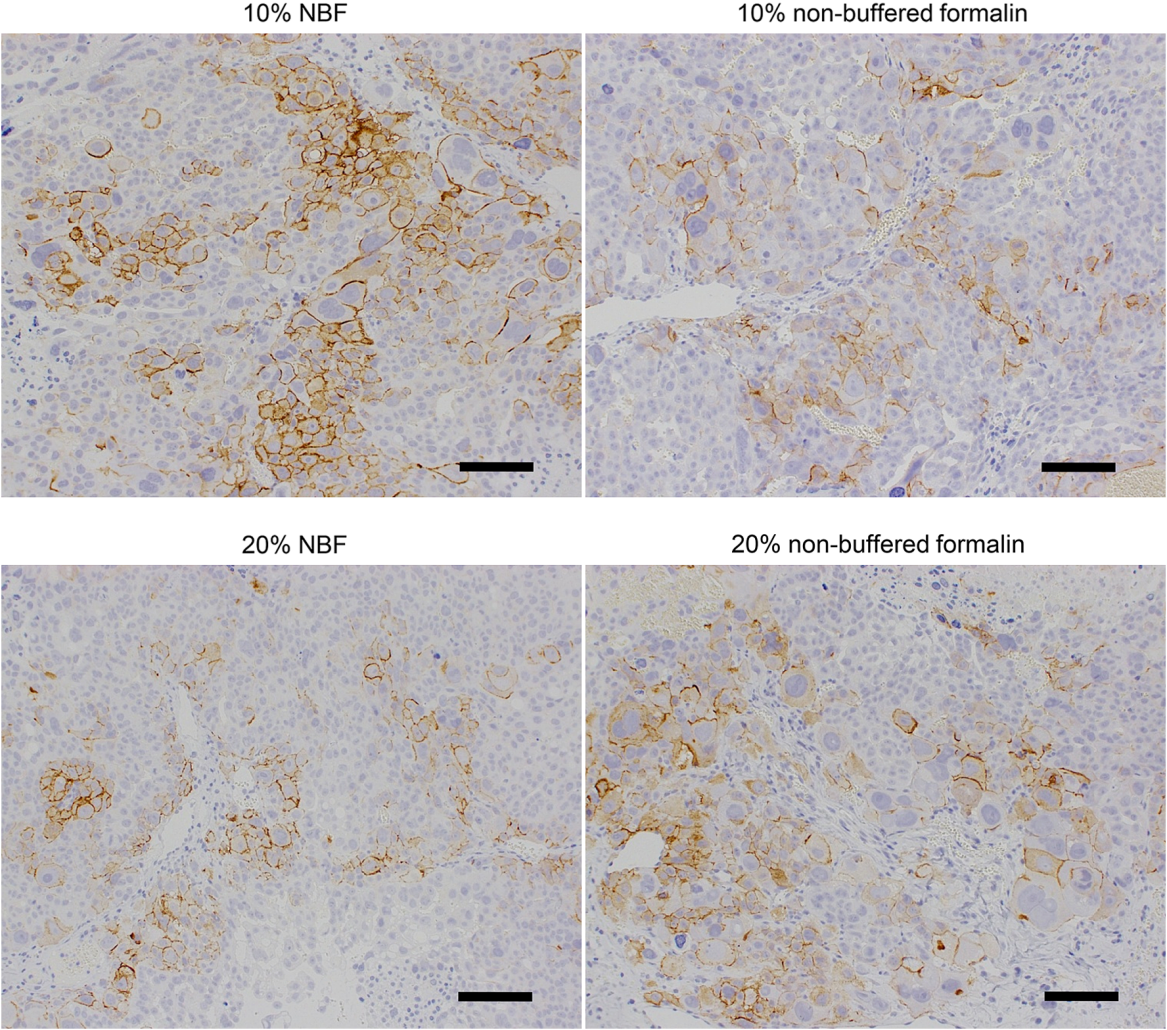
Effect of fixative type on IHC staining for HER2. SCH tumor tissues were collected and immediately fixed with 10 % NBF, 20 % NBF, 10 % nonbuffered formalin, or 20 % nonbuffered formalin for 24 h. *Bars* 100 μm

The type of fixative used for the preparation of specimens may be another important factor affecting the concordance of HER2 testing results. Therefore, we next examined the effect of fixative type on IHC staining for HER2. In this experiment, we used first specimens of the SCH tumor (HER2 IHC score 2+) because, in clinical specimens, those judged as HER2 IHC score 2+ are considered to be equivocal and the FISH test is recommended to make a final diagnosis for trastuzumab application. Therefore, it makes sense to examine whether fixative type affects the results of the HER2 IHC test in tumor tissue specimens of HER2 IHC score 2+. For this purpose, we used 10 % NBF, 20 % NBF, 10 % nonbuffered formalin, or 20 % nonbuffered formalin. The tumor tissues were fixed with each fixative immediately after collecting. Fixing with 20 % NBF, or 10 % or 20 % nonbuffered formalin for 24 h reduced the staining intensity of HER2 and reduced the HER2 IHC score from 2+ to 1+ in one of three specimens compared to fixing with 10 % NBF (Fig 4; Table 5). When tumor tissues were fixed for 10 days, the reduction in HER2 staining intensity was more apparent, and an HER2 IHC score reduction was observed in two of three specimens in 20 % NBF and 10 % nonbuffered formalin and in three of three specimens in 20 % nonbuffered formalin (Table 5). These results indicate that all formalin fixatives used, other than 10 % NBF, could negatively affect the results of HER2 IHC staining in tumors with HER2 IHC score 2+. Second, we examined the effect of fixative type on HER2 IHC staining using specimens of NCI-N87 tumor (HER2 IHC score 3+) in the same way as already described. We found that all four fixatives did not affect the results of HER2 IHC staining in either 24-h or 10-day fixation (Table 6).

**Table 5:** Effect of fixative type on HER2 IHC staining and histological assessment in SCH xenografted tumors

Fixation time	Evaluation	Fixatives			
		10 % neutral buffered saline (NBF)	10 % nonbuffered formalin	20 % NBF	20 % nonbuffered formalin
24 h	HER2 IHC score^a^	2+	1+/2+	1+/2+	1+/2+
	Histological assessment/decrease in staining^b^	–	–/+	–/+	–/+
10 days	HER2 IHC score^a^	2+	1+/2+	1+/2+	1+
	Histological assessment/decrease in staining^b^	–	–/+	–/+	+

Tests and assessments were performed in triplicate. If the test results of triplicate samples were different, all results were indicated

^a^HER2 IHC was performed by HercepTest

^b^Histological assessment on the decrease in staining was performed in terms of the following criteria and judged in a comprehensive manner: (a) autolysis, (b) decrease in positive cells, (c) decrease of total staining intensity, (d) decrease of total staining area

–, no change; ±, very slightly decreased; +, slightly decreased; ++, moderately decreased; +++, markedly decreased

**Table 6:** Effect of fixative type on HER2 IHC staining and histological assessment in NCI-N87 xenografted tumors

Fixation time	Evaluation	Fixatives			
		10 % NBF	10 % nonbuffered formalin	20 % NBF	20 % nonbuffered formalin
24 h	HER2 IHC score^a^	3+	3+	3+	3+
	Histological assessment/decrease in staining^b^	–	–	–	–
10 days	HER2 IHC score^a^	3+	3+	3+	3+
	Histological assessment/decrease in staining^b^	–	–	–	–

Tests and assessments were performed in duplicate

^a^HER2 IHC was performed by HercepTest

^b^Histological assessment on the decrease in staining was performed in terms of the following criteria and judged in a comprehensive manner: (a) autolysis, (b) decrease in positive cells, (c) decrease of total staining intensity, (d) decrease of total staining area

−, no change, ±, very slightly decreased; +, slightly decreased, ++, moderately decreased; +++, markedly decreased

## Discussion

In breast cancer, ASCO/CAP issued guidelines to standardize fixation for increased HER2 testing accuracy. In addition, many investigations into the optimal conditions for accurate HER2 testing have been conducted [15–18]. Delay to formalin fixation after specimen collection and extended fixation times may affect HER2 testing results. Several reports have shown the effect of time to fixation, fixation time, or fixative type on IHC or FISH for HER2. Khoury et al. [16] reported that more than a 1-h delay to formalin fixation negatively affected the HER2 IHC and FISH results. However, Moatamed et al. [18] showed that ischemic time (with a delay in fixation of 4 days at 4 °C), fixation time (0–168 h), and fixative type did not significantly alter HER2 IHC and FISH results. Thus, even in breast cancer, it may be considered that more studies are required to determine the most appropriate procedure for HER2 testing that can be applied in every laboratory. Needless to say, it is also important in gastric cancer to assess HER2 status accurately and reliably. However, there are no reports of analyzing formalin fixing conditions on HER2 testing in gastric cancer. In the present study, we investigated how the fixation conditions of specimens affected HER2 IHC and FISH results using paraffin-embedded gastric cell line xenografted specimens prepared using various fixation conditions. To prepare the specimens in guideline-recommended conditions, we made paraffin-embedded specimens in accordance with HER2 testing guidelines defined by the trastuzumab pathological advisory board for gastric cancer.

In the present study, leaving specimens at room temperature for more than 6 h before fixation led to shrinkage at the tumor periphery and decreased the staining intensity at the shrinkage area in all the tumors assessed, irrespective of HER2 status. However, a decrease in HER2 IHC score was observed only in SCH specimens with a 24-h delay to fixation, whereas the HER2/CEP17 ratio of FISH in SCH was decreased from 2.3 to 1.3 if the tumor sample was left at room temperature for only 6 h before fixation. In breast cancer, it has been reported that a delay to formalin fixation affects FISH results but not IHC scores [16]. Therefore, we would expect that FISH results would be more vulnerable to delayed fixation than IHC in gastric cancer specimens.

With regard to fixation time, prolonged fixation did not affect the HER2 IHC scores of NCI-N87 tumors (HER2 3+ score) (Table 2). However, in the specimens in which HER2 scores were 2+ (SCH) or 1+ (SNU-16), prolonged fixation reduced the HER2 IHC score (Table 2). These results correspond well to the report of Hashizume et al. [15], who showed that prolonged fixation affects HER2 IHC score in 2+ or 1+ breast cancer specimens. Our results suggest that considerable attention should be paid to the time elapse from excision to fixation and the fixation time of tumor tissues in gastric cancer as well as breast cancer.

The present study using several types of fixatives showed that with fixatives other than 10 % NBF IHC staining of HER2 in SCH (IHC score 2+) was reduced. We also examined the effect of fixatives in NCI-N87 (IHC score 3+); however, there were no differences in staining between fixatives. Staining of specimens that moderately express HER2 protein may therefore be affected more easily by the type of fixative used compared to staining of specimens with high expression of HER2. In the present study, we did not examine the effect of fixative type on FISH results, because, for gastric cancer, IHC testing is recommended as a first examination for HER2, followed by confirmative testing by FISH for IHC score 2+ specimens.

In the clinical setting, it is important to take unique HER2 staining characteristics into consideration when standardizing HER2 testing in gastric cancer. One such characteristic is the heterogeneity defined as less than 30 % of tumor cells staining positive or only focal staining of tumor cells [13, 19]. Gastric cancer exhibits heterogeneity in up to 30 % of HER2-positive cases [19]. The other consideration is that HER2-positive gastric carcinomas are usually of the gland-forming, intestinal type and may show incomplete, basolateral, or lateral membranous staining [20]. In the present study, we used xenografted tumors from mouse models as specimens that did not reflect the heterogeneity or other staining features of human gastric cancer. Therefore, in clinical cases, the impact of formalin fixing conditions on the results of IHC staining or FISH for HER2 may not be the same as in the present study. Further study is required in a clinical setting.

In conclusion, our results indicate that delay to fixation and length of fixation may affect the HER2 IHC score in a gastric cancer model. Nonbuffered formalin or high concentrations of neutral buffered formalin (NBF) may also affect IHC results. These results highlight that it is of critical importance to optimize sample preparation conditions for HER2 testing in gastric cancer.
