# Erratum to: Importance of formalin fixing conditions for HER2 testing in gastric cancer: immunohistochemical staining and fluorescence in situ hybridization

**DOI:** 10.1007/s10120-014-0353-3

**Published:** 2014-03-20

**Authors:** Yoriko Yamashita-Kashima, Sei Shu, Keigo Yorozu, Kaoru Hashizume, Yoichiro Moriya, Kaori Fujimoto-Ouchi, Naoki Harada

**Affiliations:** 1 Product Research Department, Chugai Pharmaceutical Co., Ltd, 200, Kajiwara, Kamakura, Kanagawa 247-8530 Japan; 2 Medical Plan Management Department, Chugai Pharmaceutical Co., Ltd., 2-1-1, Nihonbashi-Muromachi, Chuo-ku, Tokyo, 103-8324 Japan

## Erratum to: Gastric Cancer DOI 10.1007/s10120-013-0329-8

The following errors unfortunately occurred in this article.

The titles of Table 2 and Table 3 should be as follows:

**Table 2** Effect of fixation conditions on HER2 immunohistochemistry (IHC) score in human gastric cancer xenografted tumors

**Table 3** Effect of fixation conditions on the detailed histological assessment of HER2 IHC staining in human gastric cancer xenografted tumors

In addition, there is an error in Table 5. Under the column heading “Fixatives”, the first column subheading should read “10 % neutral buffered formalin (NBF)”.

